# Supplementary material for: Antioxidant Scavenging of the Superoxide Radical by Yerba Mate (Ilex paraguariensis) and Black Tea (Camellia sinensis) Plus Caffeic and Chlorogenic Acids, as Shown via DFT and Hydrodynamic Voltammetry
Source: Int J Mol Sci. 2024 Aug 28;25(17):9342. doi: 10.3390/ijms25179342 (PMC11394812; doi:10.3390/ijms25179342)
Supplement: Supplementary file 1 [file ijms-25-09342-s001.zip › ijms-3142766-supplementary.pdf]

Supplementary Material

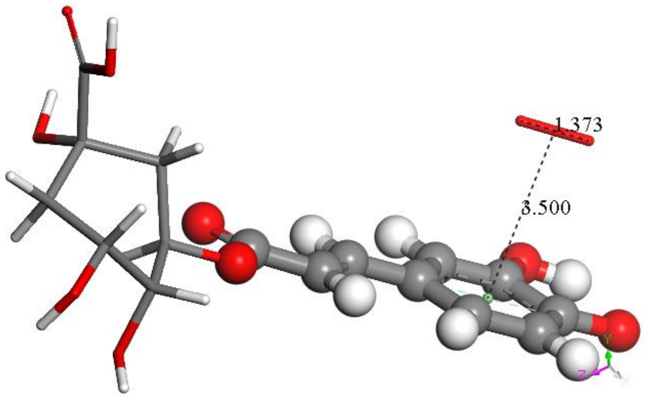

Figure S1. The initial position for a second superoxide added π-π to the chlorogenic acid ring of Figure 14 fragment.

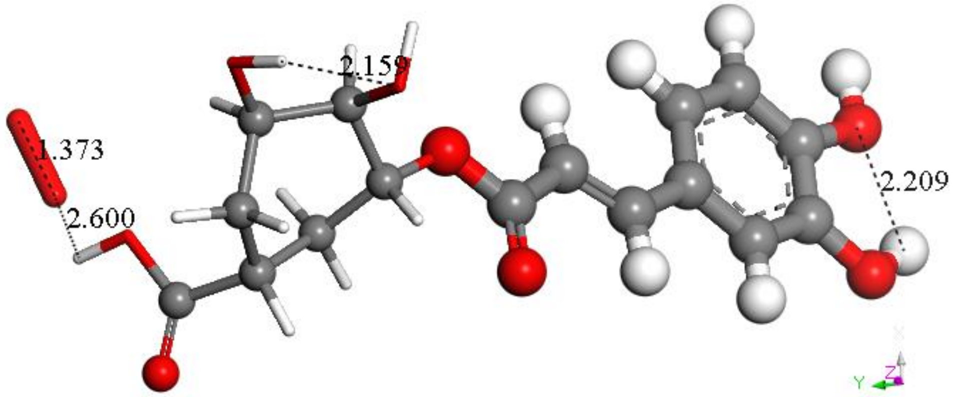

Figure S2. This is the initial arrangement for superoxide, at van der Waals distance, 2.60 Å, interacting σ style with the carboxylic moiety of chlorogenic acid.

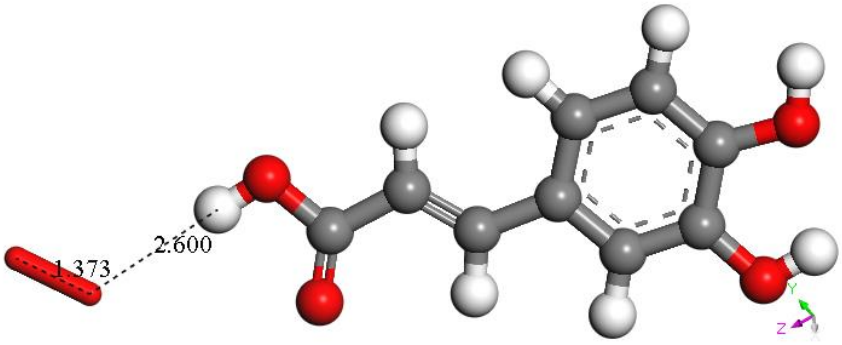

Figure S3. Superoxide is placed near the acidic proton of caffeic acid, 2.60 Å.

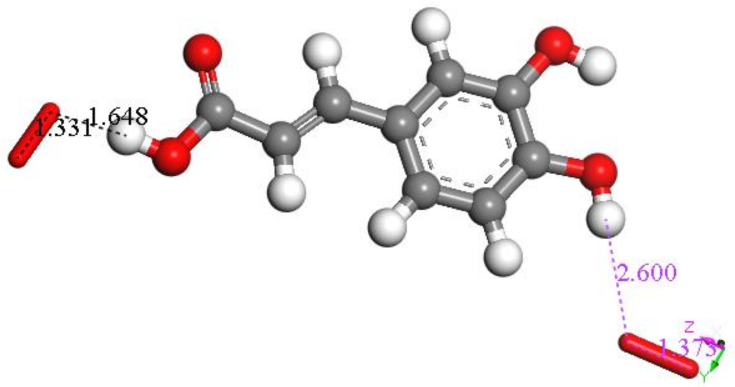

Figure S4. An additional superoxide is placed near H4 in the arrangement shown in Figure 20.

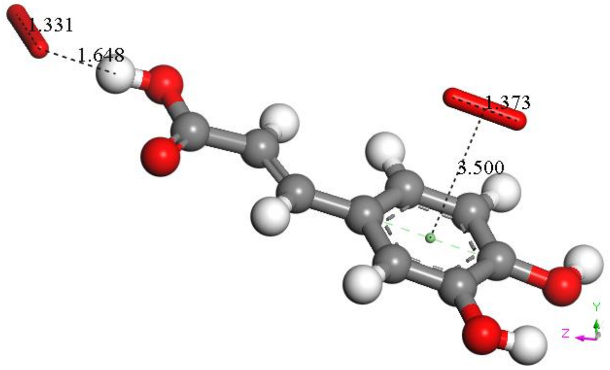

Figure S5. From the arrangement shown in Figure 20, superoxide is π-π placed above the aromatic ring. DFT minimization showed this π-π superoxide redirected towards H4 and establishing a HO_2^- anion moiety equal to Figure 21.

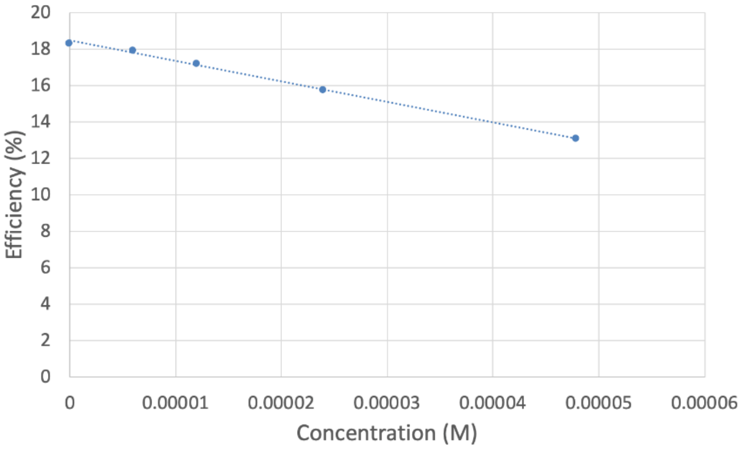

Figure S6. Linear behavior of the first 5 data (maximum = 80 μL) in the collection efficiency of caffeic acid shown in Figure 23.

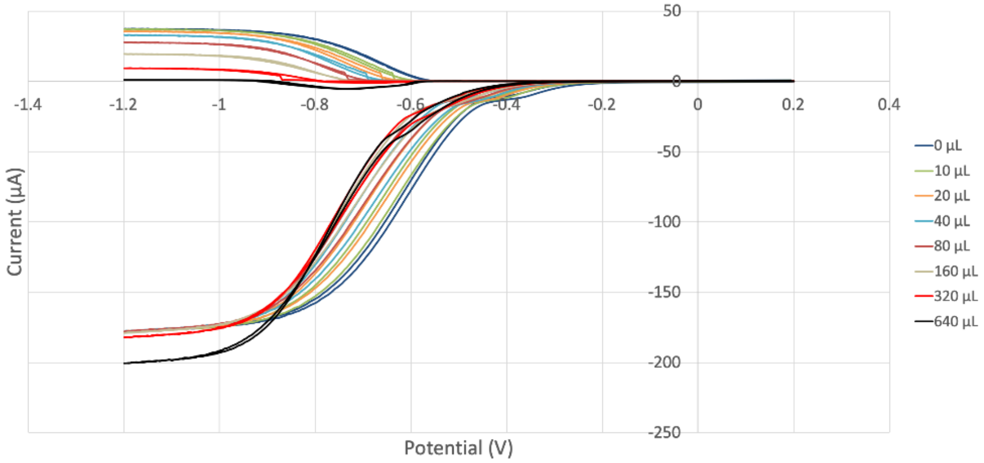

Figure S7. Voltammograms of chlorogenic acid.

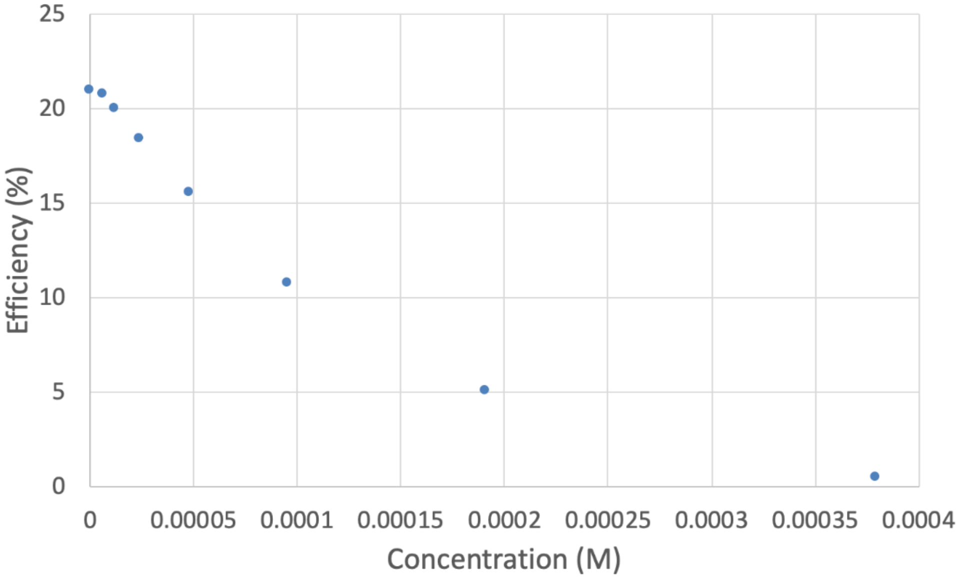

Figure S8. Collection efficiency of chlorogenic acid, the initial 5 data show a linear behavior whose linear equation is y –118,071x + 21.222 (R^2 = 0.9928). Its slope, –11.8 x 10^4, is assigned to the antioxidant capability of scavenging superoxide by chlorogenic acid.

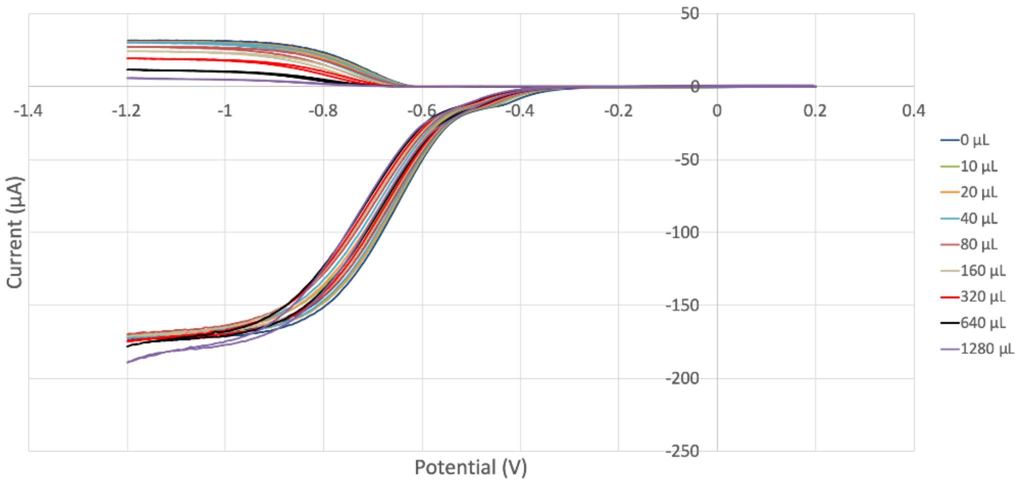

Figure S9. Voltammograms of Black tea Grade B Isphahani, each color indicates a single voltammogram experiment.

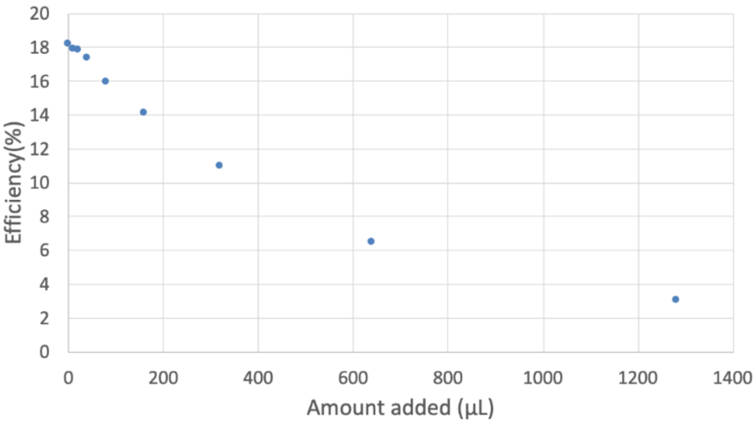

Figure S10. Collection efficiency of Black tea Grade B Isphahani. The linear behavior including the first 7 data (maximum aliquot 320 μL) shows y = -0.0230 x + 18.146 (R^2 = 0.9923). The efficiency of the last aliquot, 1,280 μL, is markedly displaced from the line, which suggests some interaction of this added tea component with a species derived from superoxide of previous aliquots.

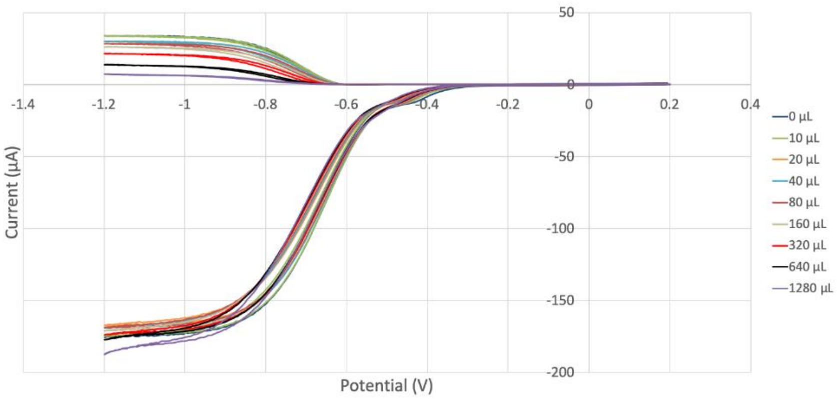

Figure S11. Voltammograms of Black Tea Grade A {Red Label}.

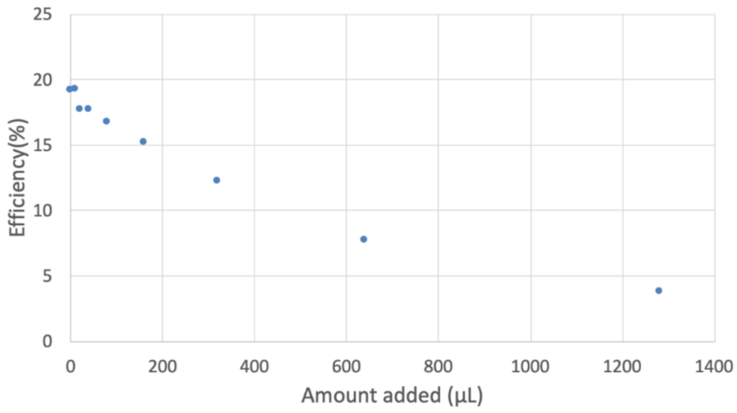

Figure S12. Collection efficiency of Black Tea Grade A {Red Label}. The linear behavior including all data (maximum aliquot 1,280 μL) shows y = -0.0118 x + 19.089 (R^2 = 0.9870). .

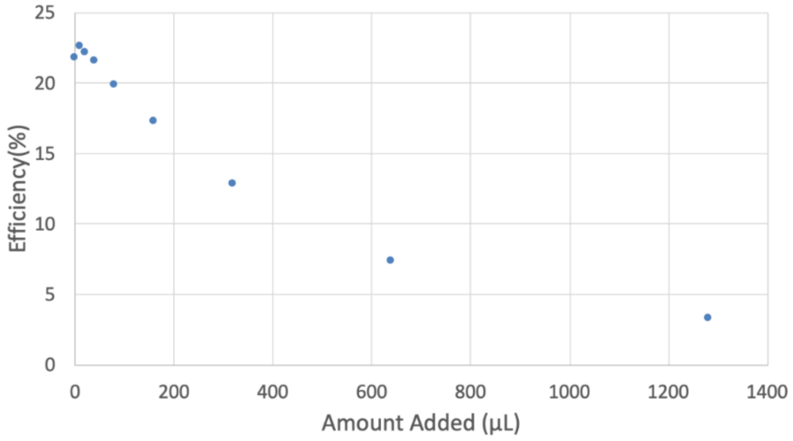

Figure S13. Collection efficiency of Pu-erh tea. The linear behavior including only 6 initial data (maximum aliquot 160 μL) shows y = -0.0322 x + 22.547 (R^2 = 0.9539).

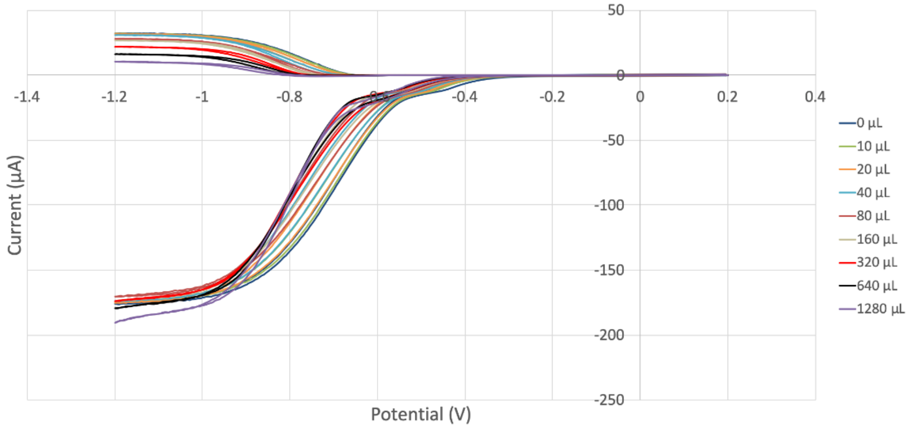

Figure S14. Voltammograms of Yerba Mate leaves and twigs, Rosamonte.

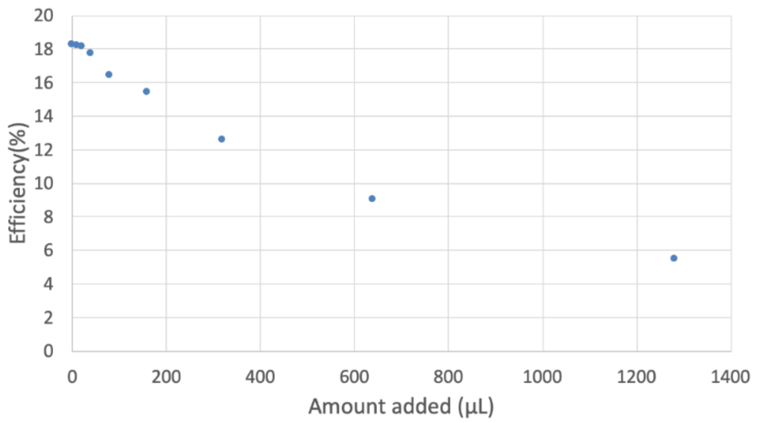

Figure S15. Collection efficiency of Yerba Mate leaves and twigs, Rosamonte. The linear behavior including only 7 initial data (maximum aliquot 320 µL) shows y = -0.0182 x + 18.317 (R^2 = 0.9898).

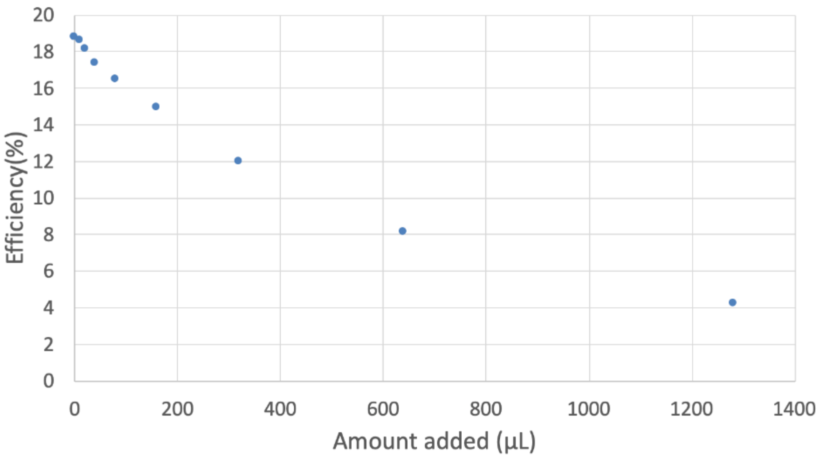

Figure S16. Collection efficiency of Yerba Mate leaves only, La Merced de Monte. The linear behavior including only 5 initial data (maximum aliquot 80 µL) shows y = -0.0239 + 18.471 (R^2 = 0.9456).
